# Genetic Analysis of a Pedigree With Antithrombin and Prothrombin Compound Mutations and Antithrombin Heterozygotes

**DOI:** 10.3389/fgene.2022.832582

**Published:** 2022-04-04

**Authors:** Haiyue Zhang, Yiling Hu, Dongli Pan, Yuehua Xv, Weifeng Shen

**Affiliations:** Department of Clinical Laboratory, The First Hospital of Jiaxing, The Affiliated Hospital of Jiaxing University, Jiaxing, China

**Keywords:** *F2*, *SERPINC1*, deep vein thrombosis, acute pulmonary embolism, subcutaneous ecchymosis, novel mutation

## Abstract

**Background and Aims:** Antithrombin (AT) is the most important physiological inhibitor *in vivo*, and coagulation factor II (FII) or prothrombin is a coagulation factor vital to life. The purpose of our research was to illustrate the connection between gene mutations and the corresponding deficiencies of AT and FII.

**Methods:** Functional and molecular analyses were performed. The possible impact of the mutation was analyzed by online bioinformatics software. ClustalX-2.1-win and PyMol/Swiss-Pdb Viewer software were used for conservative analyses and to generate molecular graphic images, respectively.

**Results:** The proband showed a lower limb venous thrombosis and acute pulmonary embolism infarction with reduced AT activity (50%). His mother, with subcutaneous ecchymosis, had reduced activities of AT and FII, of 44 and 5%, respectively. Molecular analysis showed that both the proband and his mother carried c.964A > T (p.Lys322stop) heterozygotes in *SERPINC1*. The difference was that his mother carried homozygous c.494C > T (p.Thr165Met) in *F2*, while the proband was wild type. Bioinformatics and model analysis indicated that mutations may destroy the function and structure of AT and FII protein.

**Conclusion:** This study identified a novel mutation of *SERPINC1* and a missense mutation of *F2*, which may be the molecular mechanism leading to AT and FII deficiency in this family. It will help genetic diagnosis and counseling for thrombotic families.

## Introduction

Venous thromboembolism (VTE) encompasses deep vein thrombosis (DVT) and pulmonary embolism (PE), caused by a variety of factors (Caspers et al., 2012). The pathogenesis of VTE is multifactorial, involving the interaction between clinical risk factors and thrombotic tendency, mainly including two types: hereditary and acquired. Surgery, trauma, sedentary, pregnancy, and cancer are considered acquired risk factors of VTE. Studies have demonstrated that genetic factors are responsible for more than 60% of common thrombotic susceptibility (Yue et al., 2019).

Antithrombin (AT) is a physiological anticoagulant, mainly synthesized by the liver, with a half-life of about 2.4 days (Liu et al., 2021). The mature AT molecule has 432 amino acids, including six cysteine residues that form three intramolecular disulfide bonds: Cys8-Cys128, Cys21-Cys95, and Cys247-Cys430. There are also four asparagine residues (Asn95, Asn135, Asn155, and Asn192) (Kottke-Marchant and Duncan, 2002). As a serine protease inhibitor belonging to the serine protease inhibitor superfamily, AT can inhibit activated coagulation factors II and X, and to a lesser extent activated factors IX, XI, and XII (Bafunno and Margaglione, 2010). Inherited antithrombin deficiency was first described by Egeberg in 1965 and is the main genetic factor for thrombosis, leading to a 20-fold increase in the risk of venous thromboembolism. It is found in 2–5% of patients with VTE (Rossi et al., 2008).

Prothrombin (FII, coagulation factor II) is a multidomain glycoprotein that is vital to life and an attractive target for anticoagulation therapy (Chinnaraj et al., 2018). Due to bleeding complications, mice lacking prothrombin die prematurely during the embryonic stage (Sun et al., 1998). FII is an allosteric enzyme regulated by sodium binding, controlled by five amino acid residues (Thr540, Arg541, Glu592, Arg596, and Lys599). Mutations in these residues may prevent FII from being inhibited by antithrombin, leading to continuous activation of FII, prone to thrombotic events (Tang et al., 2020). FII is synthesized by hepatocytes into a single polypeptide precursor composed of 622 amino acids. After extensive post-translational modification, FII is secreted into the plasma in its mature form and circulates in the plasma at a concentration of 0.1 mg/ml, with a half-life of about 60 h (Vostal and McCauley, 1991). Hereditary FII deficiency is an autosomal recessive inheritance with an estimated prevalence of 1:2,000,000 people. Heterozygotes with a normal *F2* gene are rarely detected clinically as FII activity (FII:C) is usually within the normal range and hardly results in any bleeding symptoms (Lefkowitz et al., 2003; Kuijper et al., 2013).

In this paper, we recruited a Chinese patient with lower limb venous thrombosis and acute pulmonary embolism infarction. Gene mutation analysis was performed to detect the patient’s genetic lesions, and finally a novel heterozygous nonsense mutation was found in the *SERPINC1* gene. It is worth noting that his mother carried the heterozygous nonsense mutation in *SERPINC1* and a homozygous missense mutation in *F2*, with subcutaneous ecchymosis.

## Case Presentation

**FIGURE 1:**
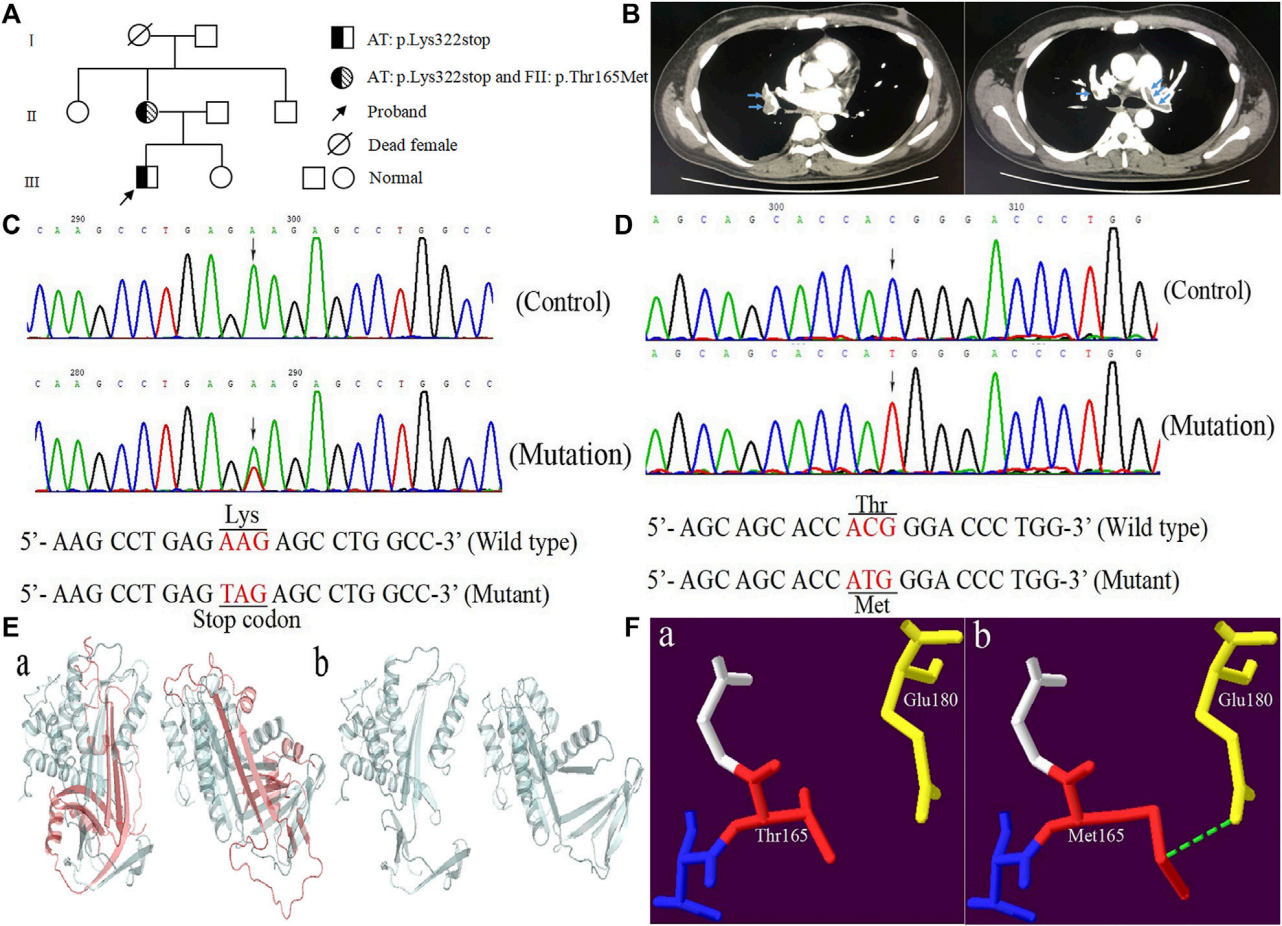
The clinical and genetic data of the proband. **(A)** Pedigree chart of the family. **(B)** The computed tomography angiography of the proband. **(C)** Sequence diagrams of *SERPINC1*:p.Lys322stop. **(D)** Sequence diagrams of *F2*:p.Thr165Met. **(E, F)** Model analysis diagrams: **(A, B)** AT:p.Lys322stop and **(A, B)** FII:p.Thr165Met before and after mutations.

A Chinese patient with lower limb venous thrombosis and acute pulmonary embolism infarction was enrolled from southeast China (Figure 1A). The proband, a 24-year-old man, presented to our hospital because of chest tightness for 8 h, feeling weak, walking unsteadily, and left thigh being thicker than before. His B-ultrasound showed enlargement of the right ventricle, moderate pulmonary hypertension, a small amount of pericardial effusion, and thrombosis in the left common iliac vein, external iliac vein, superficial femoral vein, deep femoral vein, popliteal vein, and peroneal vein. The computed tomography angiography (CTA) showed filling defects in both lung lobes and part of the arteries, leading to the consideration of pulmonary embolism (Figure 1B). To identify the possible cause of thrombosis in this patient, we conducted screening for genetic risk factors predispose to DVT, and the results showed that the proband’s antithrombin activity (AT:A) was reduced to 50% (reference range: 85∼120%), parallel decrease in antithrombin antigen content (AT:Ag) was the same as AT:A, remaining at 49 mg/dl (reference range: 80∼120 mg/dl), the anticardiolipin antibody was negative, the serum homocysteine and coagulation factor levels were normal, the activities of PS and PC were within the normal range, and blood lipids were higher (Table 1). The other secondary risk factors of thrombophilia were also ruled out. Finally, the patient was successfully treated with pulmonary angiography, inferior cavity arteriography, vascular thrombolysis, thrombus aspiration, and inferior vena cava filter implantation.

**TABLE1:** The laboratory data of the proband.

	Measure parameters	Data (proband)	Data (mother)	Reference ranges
Peripheral blood	White blood cells	7.62×10^9^/L	5.17×10^9^/L	3.97∼9.15×10^9^/L
	Red blood cells	5.11×10^12^/L	4.54×10^12^/L	4.09∼5.74×10^12^/L
	Hemoglobin	149 g/L	137 g/L	131∼172 g/L
	Platelets	373×10^9^/L	208×10^9^/L	85∼303×10^9^/L
Blood chemistry	Na	139 mmol/L	131 mmol/L	130∼147 mmol/L
	K	3.98 mmol/L	3.56 mmol/L	3.5∼5.1 mmol/L
	Cl	105 mmol/L	106 mmol/L	96∼108 mmol/L
	Total protein	82 g/L	78 g/L	60∼83 g/L
	Aspartate aminotransferase	30 u/L	34 u/L	8∼40 u/L
	Alanine aminotransferase	53 u/L	44u/L	10∼64 u/L
	Blood urea nitrogen	5.5 mmol/L	4.3 mmol/L	2.5∼7.1 mmol/L
	Creatinine	63 umol/L	65 umol/L	62∼115 umol/L
	Uric acid	478 umol/L	330 umol/L	160∼430umol/L
	Total cholesterol	6.15 mmol/L	4.5 mmol/L	2.33∼5.7 mmol/L
	Triglyceride	3.2 mmol/L	1.57 mmol/L	0.56∼1.7 mmol/L
	LDL	4.13 mmol/L	2.33 mmol/L	1.3∼4.3 mmol/L
	HDL	0.85 mmol/L	1.6 mmol/L	0.8∼1.8 mmol/L
	Apolipoprotein A1	1.3 g/L	1.58 g/L	1.06∼1.88 g/L
	Apolipoprotein B	1.39 g/L	1.03 g/L	0.46∼1.13 g/L
	Lipoprotein (a)	0.15 g/L	0.13 g/L	<0.3 g/L
	Apolipoprotein E	5.8 mg/dl	3.3 mg/dl	2.9∼5.3 mg/dl
	sdLDL-C	1.9 mmol/L	0.8 mmol/L	0.246∼1.393 mmol/L
	Free fatty acid	0.75 mmol/L	0.25 mmol/L	0.1∼0.45 mmol/L
	Troponin I	2.8 pg/ml	1.3 pg/ml	<30 pg/ml
	BNP	58.9 pg/ml	55.1 pg/ml	5∼115 pg/ml
	Glucose	5.1 mmol/L	4.1 mmol/L	3.9∼6.1 mmol/L
	C-reactive protein	8.23 mg/L	1.16 mg/L	0∼6 mg/L
Coagulation study	PT -INR	0.92	0.82	0.8∼1.2
	APTT	28.6 S	98 S	22.3∼38.7 S
	Fibrinogen	2.1 g/L	3.3 g/L	1.8∼3.5 g/L
	D-dimer	3.92 mg/L	0.23 mg/L	<0.55 mg/L
	Protein C activity	116%	110%	70∼140%
	Protein S activity	88.5%	92.2%	63∼130%
	Antithrombin activity	50%	44%	85∼120%
	Prothrombin activity	85.3%	5%	50∼150%
	FDP	12 mg/L	0∼5 mg/L	0∼5 mg/L
	LAC	1.1	<1.2	<1.2
Anti-cardiolipin antibody	IgG	—	—	
	IgA	—	—	
	IgM	—	—	

LDL, low-density lipoprotein cholesterol; HDL, high-density lipoprotein cholesterol; sdLDL-C, small dense low density lipoprotein cholesterol; BNP, N-terminal B-type natriuretic peptide precursor; PT-INR, prothrombin time international normalized ratio; APTT, activated partial thromboplastin time; FDP, fibrinogen degradation products; LAC, lupus anticoagulant.

The proband’s mother was a 45-year-old female, who discovered coincidently during the pedigree study of the proband an AT:A of 44%, AT:Ag of 46 mg/dl, and FII:C of 5%. Other parameters were normal. She was prone to subcutaneous ecchymosis, and had no thrombosis symptoms.

## Laboratory Investigations

### Subjects

The study protocol was approved by the Review Board of The First Hospital of Jiaxing and The Affiliated Hospital of Jiaxing University and the study participants gave informed consent. Whole family members (proband and six members) were enrolled and diagnosed by B-ultrasound, CTA, and laboratory examinations.

### Genetic Analysis

Genomic DNA was isolated from peripheral blood mononuclear cells using the TIANamp Genomic DNA Kit (TIANGEN, Beijing, China). All exons of *SERPINC1* and *F2* gene along with their intron-exon boundaries and untranslated regions of 3′ and 5′ were amplified by PCR with primers designed on the genomic sequences of AT and FII (GenBank accession numbers are X68793.1 and M17262.1) on a thermal cycler (ABI Thermocycler 2720; ABI, Foster City, California, United States). The PCR products were identified by 1.2% agarose gel electrophoresis, and the positive products were purified and sent to Personal Gene Technology Corporation (Shanghai, China) for direct sequencing. Sanger sequencing revealed that the proband and his mother took c.964A > T (p.Lys322stop) in exon five of *SERPINC1* (NM_000488.4) (Figure 1C). The difference was that his mother also carried c.494C > T (p.Thr165Met) in exon six of *F2* (NM_000506.5) (Figure 1D), while the proband was wild type. The novel variant was checked in 120 normal individuals.

### In-Silico and Protein Structural Analysis

Homologous sequence alignment results showed that Lys322 was not highly conserved among the homologous species. However, 43 of the 143 amino acids deleted by p.Lys322stop were highly conserved among homologous species (*Pan troglodytes*, *Macaca mulatta*, *Canis lupus* familiaris, *Bos taurus*, *Mus musculus*, *Rattus norvegicus*, *Gallus gallus*, *Xenopus tropicalis*, *Danio rerio*, and *Oryza sativa* Japonica Group). Conservative analysis showed that Thr165 was located in the highly conserved residues in the conserved region between residues 145 and 185 (Rungroj et al., 2012). The forecasting results of AT: p.Lys322stop was “disease causing” corresponding to “MutationTaster”, and the consequence of FII: p.Thr165Met was “polymorphism”. Model analysis showed that the 143 amino acid residues deletion caused by p.Lys322stop mutation had an obvious change compared with the previous protein structure (Figure 1E). For p.Thr165Met, the Thr165 was located in the kringle one domain of FII. Once substituted by Met165, the extended side chain formed another hydrogen bond with Glu180 (Figure 1F).

## Discussion

Hereditary AT deficiency is an autosomal dominant thrombotic disease that is associated with potential risk factors for the development of DVT. Even small changes in the wild-type sequence can alter the function of the gene and cause clinical manifestations (Luxembourg et al., 2011). Hereditary FII deficiency is an autosomal recessive inheritance that is related to the lower procoagulant activity. The HGMD database (http://www.hgmd.cf.ac.uk/ac/a11.php) contains more than 480 *SERPINC1* gene mutations and 72 *F2* gene mutations have been identified. According to differences in plasma activity and antigen levels, defects can be divided into two types: quantitative (type I) synthetic protein deficiency or qualitative (type II) defects.

The mutation (p.Arg197stop) of *SERPINC1* can lead to recurrent DVT, leg vein insufficiency, varicose vein resection, crural ulcers, and a family history of venous thrombosis (Michiels et al., 1995). The mutation of p.Glu271stop is associated with recurrent DVT, cerebral artery thrombosis and pulmonary embolism (Tarantino et al., 1999). In the present study, we identified a novel mutation (c.964A > T/p.Lys322stop) of *SERPINC1* in a Chinese young man with lower limb venous thrombosis and acute pulmonary embolism infarction.

The nonsense mutation (c.964A > T/p.Lys322stop) which causes Lys322 was replaced by a stop codon (UAG), resulting in the production of truncated proteins, the disappearance of the glycosylation site Asn 192, and the disulfide bond site Cys247-Cys430. The study by Michiels JJ et al. pointed out that the absence of cross-reactive substances in the patients’ plasma indicated that p.Arg197stop either prevented the formation of stable mRNA or the translated peptide was rapidly degraded (Michiels et al., 1995). It has been reported that the 13387-9delG mutation resulted in the loss of the disulfide bond between Cys247 and Cys430, impairing the secretion and stability of the truncated AT protein associated with intracellular degradation (Wang et al., 2005). Since both c.964A > T (p.Lys322stop) and 13387-9delG mutations will cause the loss of the disulfide bond between Cys247 and Cys430, we assumed that c.964A > T (p.Lys322stop) caused the reduction of AT:A and AT:Ag by the same mechanism as 13387-9delG. In addition, the truncation of the AT protein caused by c.964A > T (p.Lys322stop) resulted in the loss of the P1-P1’ (Arg393-Ser394) bond, which could not play the role of inactivating the protease.

According to the results of our family study, the proband’s mother carried c.494C > T (p.Thr165Met), which is thought to be related to Xinjiang Kazakh thrombotic disease (Ge et al., 2014) and may play a role in kidney stone disease (Rungroj et al., 2012). However, the association of this mutation with Xinjiang Kazakh thrombotic disease may be the result of the interaction of genes and complex environmental factors. The proband’s mother had a FII:C level of 5%, the association with thrombus, if there is any, is relatively weak.

The protein model analysis showed that Thr165 (an amino acid with an uncharged and polar side chain) was replaced by Met165 (an amino acid with a non-polar side chain), which resulted in the formation of another hydrogen bond with Glu180, the change in hydrogen bond-forming was likely to consequently alter protein structure and function. The c.494C > T (p.Thr165Met) substitution may affect kringle one domain glycosylation by destroying an O-glycan site (Webber et al., 2006). The kringle one domain is important in the interaction of proteins with clotting factors, and it is believed to play a role in binding mediators and regulating proteolytic activity (Patthy et al., 1984). Thus, we considered c.494C > T (p.Thr165Met) as leading to a decrease FII:C in this family. Since the pathogenicity of c.494C > T (p.Thr165Met) was not explicitly mentioned in previous reports, and some gene defects may only show functional consequences under specific conditions. We do not rule out the existence of other mechanisms that may be involved in the reduction of FII:C in this family. It should be verified by more basic experiments in the future. A somewhat puzzling finding was that the mother was homozygous while the proband was wild type. Therefore, we decided to investigate the potential cause. We found that the mother of the proband, who worked in a tannery while pregnant, was at high risk for exposure to metal salts, mainly chromates, in the tannery. Hexavalent chromium can be taken up into cells via nonspecific ionophores, causing DNA damage by generating reactive intermediates (Arslan et al., 1987). This may be the reason why the proband was wild type.

Antithrombin is an important protein that inhibits the conversion of fibrinogen by thrombin, and the reduced activity caused by gene mutation provides conditions for thrombosis. FII plays a key role in the activation of the agglutination pathway. The reduction or lack of its activity weakens the activation of the coagulation system, and the demand for antithrombin activity is no longer prominent. The commonly used anticoagulant (dabigatran) exerts an anticoagulant effect by reducing the activity of FII. Furthermore, patients with FII deficiency have clinical manifestations ranging from life-threatening spontaneous bleeding to epistaxis (Kuijper et al., 2013). AT deficiency is a high-risk factor for thrombophilia (Corral et al., 2018), and it may reduce the risk of bleeding due to FII deficiency. The simultaneous decline of FII:C and AT:A may allow physiological coagulation and anticoagulation homeostasis to be maintained.

In conclusion, in the present study, we analyzed a pedigree with antithrombin and prothrombin compound mutations and antithrombin heterozygotes, the proband had AT deficiency, and his mother had compound AT and FII deficiencies. For antithrombin deficiency, it is necessary to evaluate the blood clotting factor levels of the patient and his relatives. This reduction in antithrombin levels may give the patient an age-independent risk of thrombosis.

## Data Availability

All datasets generated for this study are included in the article.
